# Evaluation of corrective effect of 6 degree of freedom couch on setup errors in intensity modulated radiotherapy for postoperative rectal cancer patients

**DOI:** 10.3389/fonc.2023.1030599

**Published:** 2023-02-01

**Authors:** Hui Xu, Zunhao Zhang, Bo Tian, Xiongfei Li, Yunfei Bian, Xianwei Liang, Changwen Bo

**Affiliations:** Department of Oncology, The First Hospital of Hebei Medical University, Shijiazhuang, China

**Keywords:** 6 degree of freedom couch, setup errors, intensity modulated radiotherapy, rectal cancer, cancer

## Abstract

**Objective:**

To explore the corrective effect of 6 degree of freedom couch on rotation errors in intensity modulated radiotherapy (IMRT) for postoperative rectal cancer patients, further to probe into the clinical application value of 6 degree of freedom couch in radiotherapy.

**Methods:**

From January 1, 2020 to December 1, 2020, 30 patients with rectal cancer receiving postoperative intensity modulated radiotherapy in The First Hospital of Hebei Medical University were included in this retrospective study. The setup error values in all direction of patients before and after 6 degree of freedom correction were collected during each radiotherapy session.

**Results:**

In this study, a total of 382 data before and after the correction of 6 degree of freedom couch were collected. It was found that the setup errors in the Y direction gradually increased, was maximal in the third week, and then became smaller, and the setup errors in the other directions increased with the extension of radiotherapy time and reached the maximum at the 5th week. In the translation direction, the setup errors value in Z direction occurred more frequently than that in X and Y directions between the range of 0.21-0.80 cm. In the rotation direction, the setup errors value in rotation X direction occurred more frequently than that in rotation Y and Z directions between the range of 0.21°-2.99°. In addition, after the correction of the 6 degree of freedom couch in real time, the setup errors in patients were significantly reduced in all directions (P < 0.05).

**Conclusion:**

In summary, it was recommended to clinically use 6 degree of freedom couch combined with IMRT for real-time correction of placement errors in patients with rectal cancer undergoing radiotherapy. At the same time, it was necessary to observe the tumor size and body weight changes of patients on the 5th week. If necessary, radiotherapy positioning and planning should be performed in time.

## Introduction

With the continuous rise in the occurrence of rectal cancer, the incidence and mortality of rectal cancer have ranked fifth among all malignant tumors in China, with a worldwide incidence of 10.2% and a mortality rate of 9.2% (1, 2). Postoperative radiotherapy is a kind of significant therapy to prevent local recurrence in patients with advanced rectal cancer after surgery. With the progress of science, technology and equipment, intensity-modulated radiotherapy (IMRT) has become the mainstream technology of precision radiotherapy for rectal cancer (3). IMRT refers to the radiotherapy technique that adjusts the dose intensity in the radiation field according to certain requirements under the condition that the shape of each radiation field is consistent with that of the target area (4). Mechanical errors, setup errors of different body position fixation techniques, involuntary movements of patients during treatment and other factors existing in the process of radiotherapy all weaken the accuracy of radiotherapy to varying degrees, thus affecting the implementation of radiotherapy plans and weakening the control rate of tumors (5).

To improve radiotherapy accuracy, image guided radiation therapy (IGRT) has emerged. It uses on-board kV level cone beam CT (CBCT) to obtain a series of continuous images during gantry rotation, reconstruct and generate three-dimensional CT images, match with the input planning CT images, obtain the setup errors, and reduce the setup errors by online adjustment of the treatment couch (6). The treatment couch with IGRT is divided into conventional treatment couch and 6 degree of freedom couch. The conventional couch table can obtain and correct the translation errors in the three directions of X (left and right), Y (in and out) and Z (rise and fall) except for the rotation errors in these three directions. The 6 degree of freedom couch can simultaneously deal with the translation errors and rotation error (7). In addition to three-dimensional linear errors affecting the target dose, a number of studies have shown that rotational errors also have a huge impact on the accuracy and dosimetric results of treatment, so more and more attention has been paid to the analysis and correction of rotational errors (8). At present, there were few studies on 6 degree of freedom couch positioning in postoperative IMRT for rectal cancer.

Herein, this study aimed to explore the corrective effect of 6 degree of freedom couch on rotation errors in IMRT for postoperative rectal cancer patients, further to probe into the clinical application value of 6 degree of freedom couch in radiotherapy.

## Methods

### Patients

From January 1, 2020 to December 1, 2020, 30 patients with rectal cancer receiving postoperative intensity modulated radiotherapy in The First Hospital of Hebei Medical University were included in this retrospective study. This study protocol was formulated in accordance with the requirements of the Declaration of Helsinki of the World Medical Association. It was approved by the Ethics Committee of The First Hospital of Hebei Medical University, and the informed consent forms were obtained from all patients.

### Inclusion and exclusion criteria

Inclusion criteria: 1. Patients diagnosed as rectal adenocarcinoma by pathological biopsy; 2. Patients received neoadjuvant chemotherapy; 3. Patients with normal mental state and good compliance; 4. Patients signing informed consent

Exclusion criteria: 1. Patients received previous radiotherapy; 2. Patients with the existence of other pelvic surgery treatment; 3. Patients with previous history of radiotherapy; 4. Patients with other tumors; 5. Patients with expected survival time ≤6 months; 6. Patients who do not want to participate in this study.

### CT simulation positioning

Philips large bore CT simulator was used for positioning. The patient was placed in the supine position with the bladder fully filled and fixed with a thermoplastic phantom before positioning. Contrast-enhanced scans were used, and the scanning range was from the lower edge of the second lumbar vertebra to the line of 5 cm below the ischial tuberosity, and the scanning slice thickness was set to 5 mm. The positioning image was transmitted to Xio 3D intensity-modulated radiation therapy planning system (TPS), and then the target area was delineated by the radiologist.

### Delineation of target volume

According to International Commission on Radiation Units and Measurements (ICRU) Reports NO. 50 and 62 (9), target volume and organs at risk were delineated by the attending radiologist, in which the clinical target volume (CTV) was the high-risk area of the primary lesion and the regional lymphatic drainage area. The high-risk areas of primary lesions included anastomosis area, anterior sacral area, pelvic lateral wall and ischium rectal fossa. The regional lymphatic drainage volume included mesentery region, internal iliac lymph nodes, partial and external iliac lymph nodes, and obturator lymph node region. The planned target volume (PTV) was expanded 1.0 cm in the head and foot direction, 1.0 cm in the front and back directions, and 0.5 cm in the left and right directions respectively on the basis of CTV.

### Design of treatment plan

The radiotherapy plan was made by a physicist, and a total of five fields of IMRT were planned using 6 MV X-rays. Plan criteria: PTV prescribed dose was 50 Gy, 25F, 95% PTV was required to achieve the prescribed dose. Limit dose to organs at risk: pelvic V50 < 30%, small bowel V50 < 5%, Dmax < 55 Gy, femoral head V50 < 5%, Dmax < 55 Gy, and bladder V50 < 50%.

### Treatment protocol

**Figure 1:**
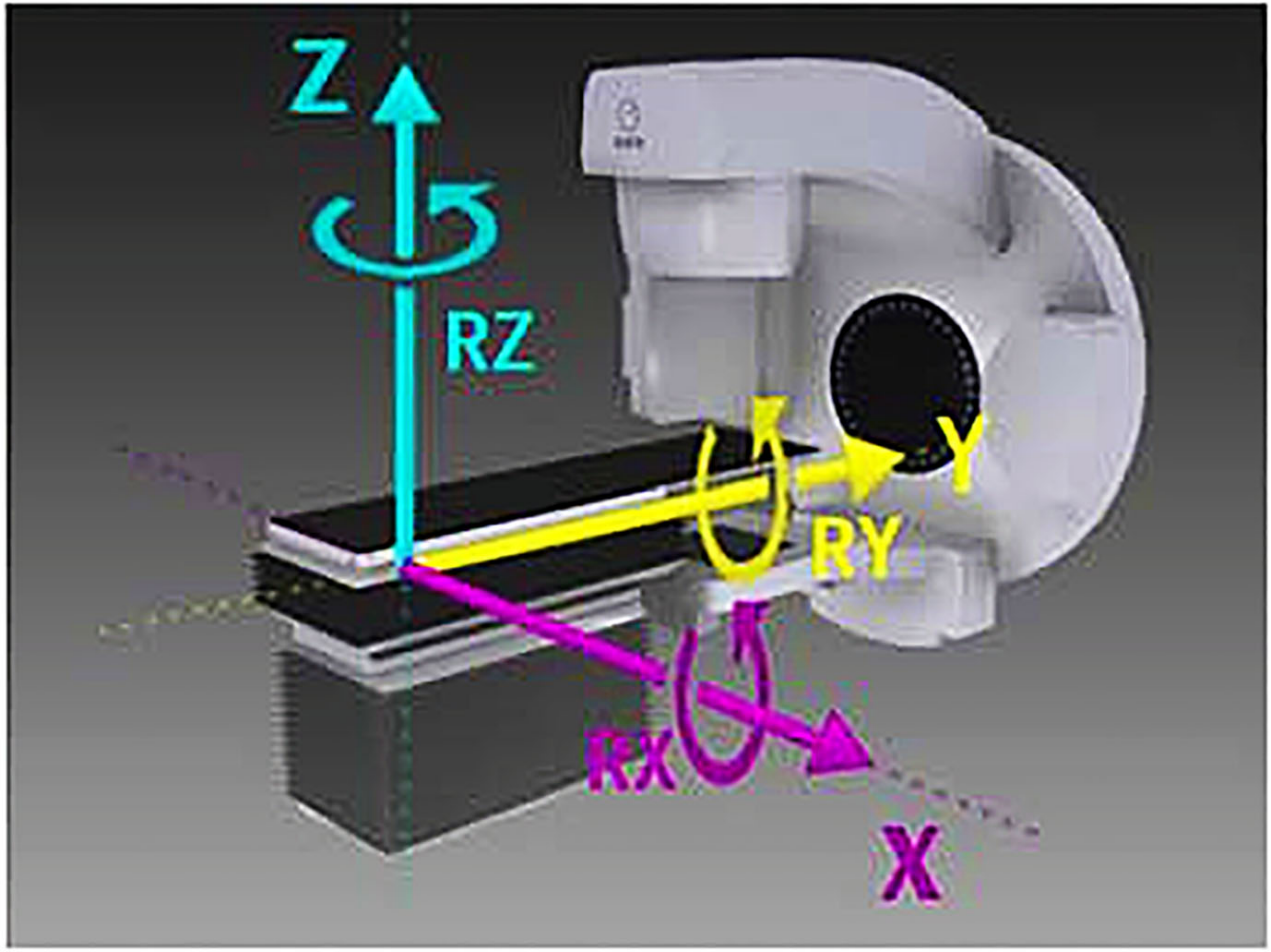
Coordinate diagram of 6 degree of freedom couch. X (left and right), Y (in and out), Z (rise and fall) and RX (rotation around X) RY (rotation around Y), RZ (rotation around Z).

All patients underwent pretreatment image guidance by linear accelerator onboard cone-beam CT before each treatment. After reconstruction, the KV CT image obtained from the scan was registered with the positioning CT image to obtain the setup errors in six directions: X (left and right), Y (in and out), Z (rise and fall) and rotation around X, Y, Z. After correcting the setup errors in real time with a 6 degree of freedom couch, a second CBCT scan was performed to obtain the residual errors of the patient and then treated (**Figure 1**). The patients were irradiated for 5 days and rested for 2 days for each session, with a total of 5 sessions.

### Statistical analysis

All the data collected in this study were analyzed using SPSS 23.0 software. The normality of continuous variables was tested by the Shapiro-Wilk test as well as the graphical illustration of histograms and Q-Q plots. Normally distributed measurement data were expressed as mean ± standard deviation (SD), while non-normally distributed measurement data were expressed as median (interquartile range), and the comparisons were examined by Student-t test and Mann-Whitney test (non-parametric distribution). The categorical data were expressed as n (%), and the differences between the two groups were examined by chi-square analysis or Fisher’s Exact Test. The statistical significance level was set at 0.05 for a two-sided test.

## Results

### Clinical characteristics

Of the 30 patients included, 19 (63.3%) were males and 11 (36.7%) were females. The age range was 45-73 years old, the average age was (57.93 ± 13.98) years. The disease course range was 3-13 months, the average disease course was (7.03 ± 1.31) months. The clinical stage was 5 (16.7%) cases of T1, 10 (33.3%) cases of T2, 9 (30%) cases of T3, 6 (20%) cases of T4. Regional lymph node status was 13 (43.3%) cases of N1, 17 (56.7%) cases of N2. The size range of primary tumor was 3-12 cm, the average tumor size was (8.23 ± 1.03) cm. The pathological type was 8 (26.7%) cases of poorly differentiated, 13 (43.3%) cases of differentiated, 9 (30%) cases of well-differentiated.

### Setup errors before the positioning correction of the couch

During radiotherapy, the setup errors in the Y direction gradually increased, was maximal in the third week, and then became smaller. The setup errors in the other directions increased with the extension of radiotherapy time and reached the maximum at the 5th week (**Table 1**).

**Table 1:** Setup errors before the positioning correction of the 6 degree of freedom couch.

Radiation weeks	Y (cm)	X (cm)	Z (cm)	Rotation X (°)	Rotation Y (°)	Rotation Z (°)
Week 1	0.16 ± 0.03	0.51 ± 0.09	-0.38 ± 0.07	0.49 ± 0.07	-0.28 ± 0.09	-0.28 ± 0.07
Week 2	0.13 ± 0.04	0.09 ± 0.03	0.34 ± 0.04	0.48 ± 0.08	-0.16 ± 0.04	-0.49 ± 0.09
Week 3	0.29 ± 0.03	0.13 ± 0.02	-0.31 ± 0.06	0.56 ± 0.09	0.07 ± 0.01	-0.35 ± 0.71
Week 4	0.13 ± 0.06	0.12 ± 0.03	-0.24 ± 0.08	0.78 ± 0.11	-0.12 ± 0.23	-0.48 ± 0.12
Week 5	0.19 ± 0.05	0.16 ± 0.02	0.21 ± 0.09	0.89 ± 0.12	0.08 ± 0.01	-0.67 ± 0.13

X direction: left and right, Y direction: in and out, Z direction: rise and fall.

### Effects of changes in bladder filling on setup errors

**Figure 2:**
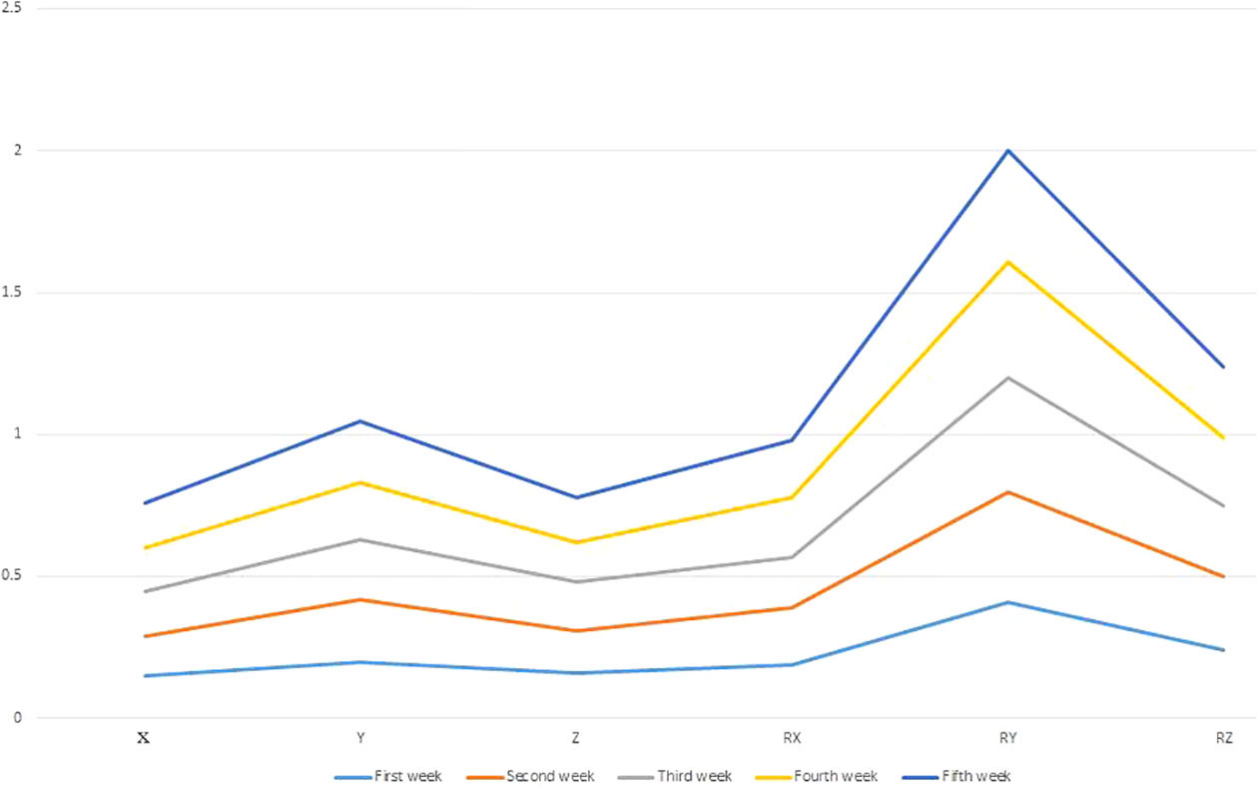
Effects of changes in bladder filling on setup errors. X (left and right), Y (in and out), Z (rise and fall) and RX (rotation around X) RY (rotation around Y), RZ (rotation around Z).

During radiotherapy, the changes in bladder filling significantly affected the setup errors, with an order of RY, RZ, Y, RX, Z and X directions, as shown in **Figure 2**.

### Setup errors distribution frequency before couch correction

In the translation direction, the setup errors value in Z direction occurred more frequently than that in X and Y directions between the range of 0.21-0.80 cm (**Table 2**). In the rotation direction, the setup errors value in rotation X direction occurred more frequently than that in rotation Y and Z directions between the range of 0.21°-2.99° (**Table 3**).

**Table 2:** Setup errors distribution frequency in translation direction before couch correction.

Distribution range of errors (cm)	Y (%)	X (%)	Z (%)
0.00-0.20	100	100	100
0.21-0.40	43	53	70
0.41-0.60	12	25	37
0.61-0.80	1	8	11
0.81-1.00	0.6	3	2
1.01-1.50	0	0.6	0.6
1.51-1.99	0	0.6	0.6
≥2.00	0	0.6	0

X direction: left and right, Y direction: in and out, Z direction: rise and fall.

**Table 3:** Setup errors distribution frequency in rotation direction before couch correction.

Distribution range of errors (°)	Rotation X (%)	Rotation Y (%)	Rotation Z (%)
0.00-0.20	100	100	100
0.21-0.50	76	77	73
0.51-1.00	53	52	49
1.01-1.50	32	25	14
1.51-2.00	22	12	8
2.01-2.50	13	6	4
2.51-2.99	6	1	3
≥3.00	0	0	0

X direction: left and right, Y direction: in and out, Z direction: rise and fall.

### Comparison of setup errors before and after couch correction

A total of 382 sets of setup errors data before and after 6 degree of freedom couch correction were collected. After the correction of the 6 degree of freedom couch in real time, the setup error in patients was significantly reduced in all directions (P < 0.05) (**Table 4**).

**Table 4:** Comparison of setup errors before and after couch correction.

	Before correction	After correction	t	P
X (cm)	0.09 ± 0.01	0.04 ± 0.01	19.365	<0.001
Y (cm)	0.17 ± 0.02	-0.04 ± 0.01	51.439	<0.001
Z (cm)	-0.31 ± 0.04	-0.05 ± 0.01	-34.539	<0.001
Rotation X (°)	0.68 ± 0.09	-0.19 ± 0.02	41.527	<0.001
Rotation Y (°)	-0.29 ± 0.04	0.42 ± 0.09	-39.485	<0.001
Rotation Z (°)	-0.39 ± 0.07	-0.07 ± 0.01	-35.631	<0.001

X direction: left and right, Y direction: in and out, Z direction: rise and fall.

## Discussion

There were many vital organs around the rectum, such as the small intestine, bladder, and femoral head. After rectal cancer surgery, the anatomical structure has changed significantly and the activity of the small intestine is limited. The bladder and small intestine were located in the horseshoe target area, so how to improve the therapeutic accuracy and reduce the exposure dose of crisis organs at risk was particularly important. IMRT was a kind of high-precision radiotherapy. In order to effectively protect the normal tissues and organs at risk around the tumor, high treatment setup accuracy was critical during the treatment (10). The inaccuracy of each setup may not only cause missed irradiation of the tumor target area, but also allow the high-dose area to move into the organ at risk area, resulting in serious complications or sequelae in patients (11). How to minimize setup errors and improve the accuracy of treatment was a hot topic in current research. IGRT was an advanced radiotherapy technology at present, and its advantage was that it combined the radiotherapy accelerator and image-guided device and corrected the setup errors in real time through image contrast. IMRT combined with IGRT technology was increasingly used for postoperative radiotherapy of rectal cancer at home and abroad (11).

Shen used CBCT to study the first setup errors of patients with pelvic tumor IMRT (including 17 patients with rectal cancer). The results showed that the application of image-guided CBCT technique could reduce the setup errors of pelvic tumor, as well as CBCT technique was helpful to determine the clinical target volume margin and reduce the side effects of treatment (12). Wu studied the setup errors between and within radiotherapy fractions for pelvic tumors and found that although there was still a slight errors after real-time correction of setup errors, it was significantly lower than the errors before correction, and the statistical results were significant, reflecting that image-guided technology improved the treatment accuracy (13). Xu used CBCT to scan the target site of 45 patients with rectal cancer during radiotherapy, established three-dimensional reconstruction images, and performed gray scale registration. After multiple image-guided corrections, the display errors were: -0.03 mm ± 0.32 mm in the left and right directions, 0.25 mm ± 0.7 mm in the head and foot directions, and -0.06 mm ± 0.66 mm in the anteroposterior direction. The results showed that the application of IGRT could reduce the setup errors and was an important quality assurance means for accurate radiotherapy (14).

In the process of radiotherapy, in addition to X, Y and Z translation errors, the patient also had errors around rotation X, Y and Z directions. It has been shown that rotation errors also affect setup accuracy as well as the exposure dose to the target area (15, 16). At present, there were few reports in the literature on the correction of setup errors by 6 degree of freedom couch combined with IGRT, and there was no study on the application of 6 degree of freedom couch in postoperative radiotherapy for rectal cancer. Boman studied 18 patients who underwent stereotactic radiotherapy (SRT) and found that if the correction of X, Y, and Z three-dimensional translation errors was performed, the appropriateness index of the treatment plan was 0.78 and 78% of the prescribed dose to the target area, and after six-dimensional correction of translation and rotation errors, the appropriateness index of the treatment plan increased to 0.91 and the target area dose also increased to 92.1% of the prescribed dose, so the correction of rotation errors was considered to be important in clinical application (17). Previous studies have shown that in the late stage of IGRT for esophageal cancer, the X and Z axial setup errors of patients became larger, and it was recommended to replace the phantom for simulated positioning from the 5th week and remake the treatment plan (18). The results of this study showed that the setup errors in translation directions increased with the extension of radiotherapy time and reached the maximum at the 5th week except for the Y direction, which was consistent with the above study results. The setup errors in the Y direction gradually increased, was maximal in the third week, and then became smaller. The reason may be that the increased setup errors was related to the decreased patient tolerance and cooperation, and the weight change and tumor size change of the patient during radiotherapy will affect the setup errors. Therefore, during the treatment, the therapist shall provide the health education for the patient emphasizing radiotherapy, so as to improve the accuracy of treatment.

Rotational errors in pelvic radiotherapy can reach a maximum of 14° (19). AhmadAhmad used common couch correction in 15 patients undergoing pelvic radiotherapy, and the maximum translational errors was 0.5 cm and the maximum rotation angle was up to -7.3°– 10°in the rotation X direction wchich was the largest in the errors in the rotational directions (20). The results of this study showed that the setup errors in all directions were significantly reduced after correction of the 6 degree of freedom couch in real time. Among the errors in the translation direction, the Y direction is the largest. Considering that the body surface positioning line changes due to the patient’s skin traction during setup, resulting in that the target area in the body does not match the body surface positioning line, so the therapist should let the patient’s buttock lift and fall naturally and reposition. In the rotation errors, the error in rotation X direction was more than that in other directions, which may be caused by the natural subsidence of the six-degree-of-freedom bed.

Previous study showed that, although the translation errors was effectively controlled in 25 patients undergoing pelvic radiotherapy after IMRT combined with three-dimensional couch correction, the residual rotation errors was still large, and it was necessary to rescan the positioning and remake the radiotherapy plan (21). The translation and rotation errors can be accurately corrected with a 6 degree of freedom couch (7). The results of this study showed that, in the translation direction the setup errors value in Z direction occurred more frequently than that in X and Y directions between the range of 0.21-0.80 cm. In the rotation direction, the setup errors value in rotation X direction occurred more frequently than that in rotation Y and Z directions between the range of 0.21°-2.99°. This results suggested that the setup errors in the six directions was significantly reduced after correction. Since it can be speculated that the setup correction of 6 degree of freedom couch combined with IMRT can further improve the radiotherapy accuracy and ensure the radiotherapy efficacy.

This study has some limitations. First, the small sample size may weaken the generalisability of the results, and further study was needed to confirm it at a larger scale. Second, this is a retrospective study. Prospective studies should be conducted in the future to further confirm the conclusions in this study. Third, there are many influencing factors of setup errors of 6 degree of freedom couch, such as weight change of patients during radiotherapy, degree of bladder holding and other factors are not included in the consideration. The relevant influencing factors should be analyzed at the next study, so as to comprehensively analyze the effect of setup errors.

## Conclusion

In summary, it was recommended to clinically use 6 degree of freedom couch combined with IMRT for real-time correction of placement errors in patients with rectal cancer undergoing radiotherapy. At the same time, it was necessary to observe the tumor size and body weight changes of patients on the 5th week. If necessary, radiotherapy positioning and planning should be performed in time.

## Data availability statement

The original contributions presented in the study are included in the article/supplementary material. Further inquiries can be directed to the corresponding author.

## Ethics statement

The studies involving human participants were reviewed and approved by The First Hospital of Hebei Medical University. The patients/participants provided their written informed consent to participate in this study.

## Author contributions

HX and CB contributed to the conception and design of the study; ZZ and BT performed the experiments, collected and analyzed data; XFL, XWL and YB wrote the manuscript; HX and CB revised the manuscript. All authors contributed to the article and approved the submitted version.
